# Synthesis and characterization of Zr- and Hf-doped nano-TiO_2_ as internal standards for analytical quantification of nanomaterials in complex matrices

**DOI:** 10.1098/rsos.171884

**Published:** 2018-06-06

**Authors:** Laura-Jayne A. Ellis, Anastasios G. Papadiamantis, Stefan Weigel, Eugenia Valsami-Jones

**Affiliations:** 1 School of Geography, Earth and Environmental Sciences, University of Birmingham, Edgbaston, Birmingham B15 2TT, UK; 2 RIKILT – Wageningen UR, Akkermaalsbos 2, 6708 WB Wageningen, The Netherlands

**Keywords:** nanosafety, nanomaterials, reference materials, hydrolysis/oxidation methods, hydrothermal, labelled titania and zirconia

## Abstract

The reliable quantification of nanomaterials (NMs) in complex matrices such as food, cosmetics and biological and environmental compartments can be challenging due to interactions with matrix components and analytical equipment (vials and tubing). The resulting losses along the analytical process (sampling, extraction, clean-up, separation and detection) hamper the quantification of the target NMs in these matrices as well as the compatibility of results and meaningful interpretations in safety assessments. These issues can be overcome by the addition of known amounts of internal/recovery standards to the sample prior to analysis. These standards need to replicate the behaviour of target analytes in the analytical process, which is mainly defined by the surface properties. Moreover, they need to carry a tag that can be quantified independently of the target analyte. As inductively coupled plasma mass spectrometry is used for the identification and quantification of NMs, doping with isotopes, target analytes or with chemically related rare elements is a promising approach. We present the synthesis of a library of TiO_2_ NMs doped with hafnium (Hf) and zirconium (Zr) (both low in environmental abundance). Zirconia NMs doped with Hf were also synthesized to complement the library. NMs were synthesized with morphological and size properties similar to commercially available TiO_2_. Characterization included: transmission electron microscopy coupled with energy-dispersive X-ray spectroscopy, X-ray diffraction spectroscopy, Brunauer–Emmett–Teller total specific surface area analysis, cryofixation scanning electron microscopy, inductively coupled plasma optical emission spectroscopy and UV–visible spectrometry. The Ti : Hf and Ti : Zr ratios were verified and calculated using Rietveld refinement. The labelled NMs can serve as internal standards to track the extraction efficiency from complex matrices, and increase method robustness and traceability of characterization/quantification.

## 1. Introduction

Titanium dioxide (TiO_2_, also known as titania) nanomaterials (NMs) below 100 nm have gained increased interest for their incorporation into cosmetic products [1] and other industrial products (e.g. paints and surface coatings) [2], particularly the rutile and anatase crystalline structures. The use of TiO_2_ NMs is based on their photoelectronic properties and their ability to photoactivate with UV radiation and act as antibacterial and/or antifouling agents in windows, pavements, walls and roofs [2], or block sunlight when used in cosmetics and help formulate a sun protection factor [1,3]. The type of TiO_2_ NM (rutile and anatase) to be used in each case is defined from their crystal structure, as anatase NMs present higher density and lower refractive index and are more photoactive than rutile NMs [4] (although these properties can be modified either through the production process or intentionally through the synthesis protocol used) [4,5]. As a result, anatase NMs are mainly used in infrastructure applications, while rutile NMs are used in sunscreens and cosmetics.

However, nanoscale properties have been linked to potential increased toxicity [6] and, therefore, nanoparticle inclusion in sunscreens has prompted research to identify potential health hazards and/or environmental impacts from their use [7]. This is particularly important because increasing evidence supports that nano-TiO_2_ properties differ substantially from those of their larger counterparts [8]. TiO_2_ is a photocatalyst and upon excitation can produce hydroxyl radicals and other reactive oxygen species (ROS) that can lead to DNA damage [1], although this same attribute is desirable when TiO_2_ NMs are used as antifouling coatings, e.g. in windows [9]. At the same time, the presence of dopants inside the TiO_2_ crystal structure can lead to undesirable effects through potential dissolution and the release of Ti^4+^ and other metals. Such releases may be potentially hard to identify, especially when Ti^4+^ and the potential dopants are naturally occurring elements (e.g. Ag). The International Agency for Research on Cancer (IARC) has classified TiO_2_ as a group 2B possible carcinogen to humans for inhalation exposure [10]. Irrespective of risk, European legislation, as well as that of other jurisdictions including the USA, regulates that the maximum concentration of nano-TiO_2_ that can be added to sunscreen products is 25% weight [3,11]. In the European Union (EU), since 2013 the use of nanosized ingredients in cosmetic products must be indicated on the product label [12]. Despite restrictions, with recreational uses, rutile TiO_2_ NMs will inevitably be dispersed into the environment [13,14]. Additional environmental release will also come from the widespread use of larger-scale pigment grade titania (median size approx. 200 nm) in paints, and anatase-based nanocoatings on external walls of buildings (facades, bricks and concrete), pavements and windows caused by environmental weathering [15,16].

It is essential that the potential exposure routes, kinetics, environmental fate and behaviour of manufactured NMs are fully evaluated and can be monitored if necessary [17]. This knowledge can be used for the risk assessment of nano-TiO_2_ and NMs in general to study and potentially decrease hazardous effects on human, animals and the environment in general. This knowledge can then be applied in safer-by-design (SbD) principles and the design and production of more stable and less hazardous TiO_2_ nanoproducts. To do so, relevant reference materials are needed that include a variation of sizes and surface functionalization for *in vitro*, mesocosm and environmental studies, which are currently absent from the literature [18]. TiO_2_ is highly abundant in the environment and can be found in forms chemically and structurally indistinguishable from its engineered counterparts [19], while the same is true for potential dopants used to modify their physico-chemical and photoelectronic properties [20]. Thus, there is urgency for manufactured TiO_2_ to be produced in a safer form, which also allows it to be identified against an environmental background. Therefore, doped titania particles, with modified photocatalytic properties, can be used as tracers to improve identification method robustness, and increase quantification, reliability and traceability by being distinguishable from their natural counterparts.

The EU FP7 project NanoDefine was launched in 2013 and ended in October 2017. The project aimed to develop cost-efficient screening methods and suitable reference materials to support the implementation of the EU Recommendation on the Definition of Nanomaterial [21–23]. More information can be found about the project at www.nanodefine.eu. In 2014, the NanoDefine consortium identified the need for more validated information on standard reference materials [21]. To address the absence of suitable reference materials, BAM (in partnership with NanoDefine) now provides an expanding database of ‘Nanoscaled Reference Materials’, accessible via: http://www.nano-refmat.bam.de/en/ in cooperation with the International Organization for Standardization (ISO)/TC 229 Nanotechnologies.

In partnership with the NanoDefine consortium, we obtained cosmetic materials and samples of powder TiO_2_, known to be used in commercial cosmetic and industrial products, which could be released into the environment, for characterization. The intention was to obtain the morphology, size and physico-chemical properties of the TiO_2_ NMs as a foundation to allow production of suitable reference materials with systematic variation of their physico-chemical properties. In addition, the challenges of extracting of TiO_2_ NMs from cosmetics for characterization were also explored using different sample extraction methods. Characterization was carried out by transmission electron microscopy (TEM) coupled with energy-dispersive X-ray spectroscopy (EDS), X-ray diffraction (XRD) analysis and Brunauer–Emmett–Teller (BET) surface area analysis.

In this paper, we present results from the development and optimization of two synthesis methods, hydrolysis/oxidation and hydrothermal synthesis, aimed at producing a library of titania NMs with a range of surface treatments (including alumina, acetate and stearate) and doping agents (including hafnium (Hf) and zirconium (Zr)). These methods are reproducible and the final products mimic particles/materials commercially available for use in industrial, sunscreen and other cosmetic products. Moreover, because Zr and Hf are chemically similar to titanium (Ti), the oxides of these elements may potentially be synthesized using the same protocols as TiO_2_ [24,25] or incorporated into TiO_2_ as dopants. At the same time, Zr and Hf NMs present either similar [26] or lower [27,28] toxicity than TiO_2_ NMs during *in vitro* experiments, respectively. Given their lower abundance in the environment compared with Ti [29], they could be used as tracers and assist the development of relevant risk assessment protocols. We also synthesized ZrO_2_ NMs doped with Hf as an additional reference material that could serve as a complement to nano-TiO_2_ tracers in environmental samples and for environmental fate studies.

## 2. Material and methods

### 2.1. Materials

All chemicals and solvents were purchased from Sigma-Aldrich (Dorset, UK) and were of analytical reagent grade. Ultra-pure water (UPW) with a maximum resistivity of 18.2 MΩ · cm was used throughout the experiments.

### 2.2. Extraction of TiO_2_ from sunscreen products and TiO_2_ nanopowders

To produce suitable labelled TiO_2_ particles, the NanoDefine consortium provided two cosmetic samples termed BAM-13A and BAM-13B, as cited in their Technical Report D2.4 [22] for characterization purposes and to serve as model materials. Sample BAM-13A is a complete formulated sample containing 4% nano-TiO_2_ with an aluminium (Al) salt-based surface coating, micro-TiO_2_ and iron oxides (for colouring purposes). Sample BAM-13B is a simplified formula containing only the 4% nano-TiO_2_ (same particles as BAM-13A), with the Al salt-based surface coating [22]. Prior to characterization, the TiO_2_ NMs were removed from the formulated samples using a solvent extraction process. Briefly, 3 g of each sample was mixed with 15 ml of either ethanol, methanol or acetone to remove the cosmetic matrix (such as emulsifiers, paraffin and silicones). The dispersions were vortex mixed; this was followed by centrifugation (5000 r.p.m. for 20 min). The pellet was resuspended with the solvent and the process was repeated three times to ensure all organic components were removed, as reported in the literature [30]. Finally, the solid pellet was removed and left to air-dry for 24 h to obtain the metal oxide powder for characterization. In addition to the samples provided by the NanoDefine consortium and in order to maximize the relevance of the work, a number of samples of TiO_2_ powders for commercial use were obtained for characterization from www.ulprospector.com. These included:

— *TTO NJE8*. TiO_2_, alumina and jojoba esters.
— *JTTO MS7*. TiO_2_, alumina and methicone.
— *UV Balance Powder 100 NJE8*. TiO_2_, alumina and jojoba esters.
— *TiO_2_ TA-100*. TiO_2_, alumina and silica.

All obtained samples were characterized to identify size, composition, morphology and surface area, and were subsequently used as a guide for the in-house synthesized TiO_2_ and ZrO_2_ reference NMs. For reference, these samples will be referred to as the commercial TiO_2_ powders for the rest of the paper.

### 2.3. Characterization of TiO_2_ NMs

To assess composition, size, crystalline structure and purity, complete and simple formula samples (NanoDefine), commercial TiO_2_ powders and in-house synthesized TiO_2_ and ZrO_2_ reference NMs were characterized by means of TEM, EDS, XRD, BET, scanning electron cryomicroscopy (cryo-SEM) and inductively coupled plasma optical emission spectroscopy (ICP-OES).

TEM analysis was performed using a JEOL 1200EX 80 kV Max system. All sample dispersions were prepared by suspending 0.01 g of the TiO_2_ solids into 5 ml UPW. TEM grids were prepared by a drop-casting method, whereby a 20 µl drop of TiO_2_ suspension was deposited on a 300 mesh carbon-coated copper TEM grid (Agar Scientific, UK). The TiO_2_ suspension drop was left for approximately 30 min to allow the NMs to adhere to the carbon membrane, and grids were rinsed with UPW to remove excess water to avoid aggregation. EDS analysis was carried out using a JEOL 2100 TEM fitted with an Oxford Inca EDS, with an accelerating voltage of 200 kV. Particle diameter measurements were conducted using the Gatan Digital Micrograph software by measuring at least 100 particles.

Cryo-SEM was used to image the NanoDefine samples in the original formulated products to avoid any drying artefacts from solvent extraction methods. Imaging was conducted using a Philips XL-30 FEG ESEM coupled with an HKL Channel 5 electron backscatter diffraction detector and an Oxford Inca X-ray EDS detector. Samples of the complete and simple formula (BAM-13A and BAM-13B) were frozen under liquid nitrogen on the cold stage inside the instrument and were etched to reveal the particles inside the emulsion layers of the formulae.

Crystalline phases were identified for all dispersions using a Bruker D8 autosampler powder diffraction XRD system and the data were analysed using the Eva software. Scans were performed at 2*θ* between 20° and 60°. The presence of dopants and ratios between Ti and either Hf or Zr and Zr and Hf were verified and calculated through Rietveld refinement of the XRD diffractograms using the GSAS II software [31]. Owing to the low crystallinity of the diffractograms, high-quality phases were acquired from the FIZ Karlsruhe Inorganic Crystal Structure Database for the rutile [32] and anatase [33] TiO_2_ and monoclinic ZrO_2_ [34]. Those phases were used to calculate the unit cell dimensions, volume and density, and the atomic coordinates of the elements present inside the undoped reference phases. The latter were then used to verify the presence of dopants in the respective phases and calculate the unit cell parameters, atomic coordinates and the percentage of doping of the doped phases (Ti*_x_*Hf*_y_*O_2_, Ti*_x_*Zr*_y_*O_2_ and Zr*_x_*Hf*_y_*O_2_).

Specific surface area analysis using BET was conducted on all samples (except the complete and simple formulated NanoDefine samples due to lack of sufficient material) using a Beckman Coulter SA 3100 instrument. Briefly, the TiO_2_-containing powders were weighed within the ranges of 0.2–0.4 g and were outgassed for 280 min prior to analysis at a furnace temperature of 300°C. Next, using liquid nitrogen, the volume of gas adsorbed to the particle surface was measured to give the total surface area (m^2^ g).

A Jenway 6800 double beam UV–visible spectroscopy (UV–vis) was used to measure the absorbance of the TiO_2_ NM suspensions. The absorption peak was also used to calculate the energy band gap of the studied NMs using Tauc Plots [35]. This was achieved using the right shoulder of the absorption peak for which linear fitting was performed to calculate the cut-off wavelength (*λ*, the wavelength where the fitting crosses the horizontal wavelength axis) and the absorbance reaches a minimum. This energy band gap is then calculated using the equation *E* = *h *×* C*/*λ*, where *h* is Planck's constant (6.626 ×* *10^−34^ J s), *C* is the speed of light (3.0 × 10^8^ m s^−1^), *λ* is the cut-off wavelength in nm and 1 eV = 1.6 × 10^−19^ J (conversion factor).

The concentration of the in-house synthesized TiO_2_ series NMs was measured on a Perkin Elmer Optima 8000 ICP-OES using the Syngistix Software. Samples of 0.01 g were prepared using an aqua regia digest (2%) 24 h prior to analysis and calibrated with TiO_2_ standards from 0 to 100 mg l^−1^.

### 2.4. Reference NM synthesis

#### 2.4.1. Alumina-coated TiO_2_

Initial TiO_2_ synthesis was based on revising the approaches by Cassaignon *et al.* [36] and Valsami-Jones *et al.* [7], which involved a traditional hydrolysis and oxidation method. A titanium (III) chloride (TiCl_3_) solution in 12% hydrochloric acid (HCl) was introduced to UPW under vigorous stirring, to produce a final solution strength of 0.15 M and the final solution pH was adjusted to pH 4. The dispersions were heated at 60°C on a hot plate until the violet solution became a white precipitate. Solids were washed and obtained by centrifuging the precipitate with UPW (to remove excess salts) at 5000 r.p.m. for 20 min. The washing procedure was conducted three times to ensure all excess salt solutions were removed, and the remaining solid was dried at 60°C for 6 h. The TiO_2_ particles were then film-coated according to a modified protocol based on Wu *et al.* [37]. Briefly, a stock solution of 0.4 g of synthesized TiO_2_ was suspended in 100 ml of UPW and sonicated for 2 h. Twenty-five millilitres of the stock solution was diluted 50 : 50 under vigorous stirring conditions. A 0.03 mol l^−1^ solution of aluminium chloride hydrate (AlCl_3_) was added by titration to the TiO_2_ suspension, and the pH was adjusted again to pH 4 using a 0.1 mol l^−1^ NaOH solution. After vigorous mixing, the dispersions were heated to 60°C for 2 h and centrifuged at 5000 r.p.m. for 30 min to obtain a solid white pellet. The solids were oven-dried at 60°C for 12–24 h [37].

#### 2.4.2. Hafnium- and zirconium-doped TiO_2_

It was not possible to synthesize good-quality doped TiO_2_ NMs using the hydrolysis and oxidation method as described above. This method did not produce homogeneous size/structures or morphologies of the desired particles in question nor did it incorporate the dopant into the metal oxide core of the NMs. Alternative hydrothermal methods were therefore considered to complement the synthetic metal oxide NM library [38–41]. After trials, the synthesis of a range of doped NMs was achieved by hydrothermal synthesis using Ti (IV) isopropoxide, as the precursor, and a 0.01% molar ratio of Hf (IV) chloride (HfCl_4_) or Zr (IV) oxynitrate hydrate (ZrO(NO_3_)_2_ · *x*H_2_O) as the doping agents in an alcohol aqueous dispersion. All dispersions were subject to hydrothermal treatment, which used Teflon-lined autoclave vessels (Parr Instruments) maintained at 200°C for 24 h. After hydrothermal treatment, the autoclave vessel was cooled at room temperature before the precipitate was removed, washed with UPW and centrifuged at 5000 r.p.m. for 30 min. The remaining precipitate was oven-dried at 60°C to obtain a white powder containing the Hf- or Zr-doped titania particulates. The drying procedure was repeated for all particles synthesized by hydrothermal treatment including NMs described in §2.4.3.–2.4.6.

#### 2.4.3. Hafnium-doped TiO_2_ with acetate surface treatment

A 4 ml solution of Ti (IV) isopropoxide was added to a solution containing 17 ml of benzyl alcohol, 3 ml of 0.013 acetic acid and 10 ml of 0.01 M HfCl_4_. The dispersion was mixed together for 30 min before hydrothermal treatment (§2.4.2.).

#### 2.4.4. Hafnium-doped TiO_2_ with stearate surface treatment

A 4 ml solution of Ti (IV) isopropoxide was added to a solution containing 17 ml of benzyl alcohol, 3 ml of 0.013 M stearic acid and 10 ml of 0.01 M HfCl_4_. The dispersion was mixed together for 30 min before hydrothermal treatment (§2.4.2.).

#### 2.4.5. Zirconium-doped TiO_2_ without surface treatment

A 4 ml solution of Ti (IV) isopropoxide was added to a solution containing 17 ml of benzyl alcohol and 13 ml of 0.01 M ZrO(NO_3_)_2_ · *x*H_2_O. The dispersion was mixed together for 30 min before hydrothermal treatment (§2.4.2.).

#### 2.4.6. Hafnium-doped ZrO_2_ without surface treatment

In addition to the traditional TiO_2_ synthesis, ZrO_2_ NMs were also synthesized as pure ZrO_2_ and with an Hf-doping agent to produce reference materials similar to the TiO_2_ NMs. A starting solution of 0.5 M Zr (IV) oxynitrate hydrate (ZrO(NO_3_)_2_ · *x*H_2_O) and the doping agent of 0.01 M HfCl_4_ were prepared in 10 ml UPW and stirred vigorously to mix. A 5 M solution of NaOH (10 ml) was added to the dispersion at a rate of 1 drop per second to produce a white precipitate. Once all the NaOH was added, the mixture was stirred for a further 10 min and sonicated for 1 h. After sonication, 4 ml of ethanol was added to the mixture and stirred for 5 min before hydrothermal treatment (§2.4.2.).

## 3. Results and discussion

### 3.1. Characterization of the complete formula sunscreen

**Figure 1.**
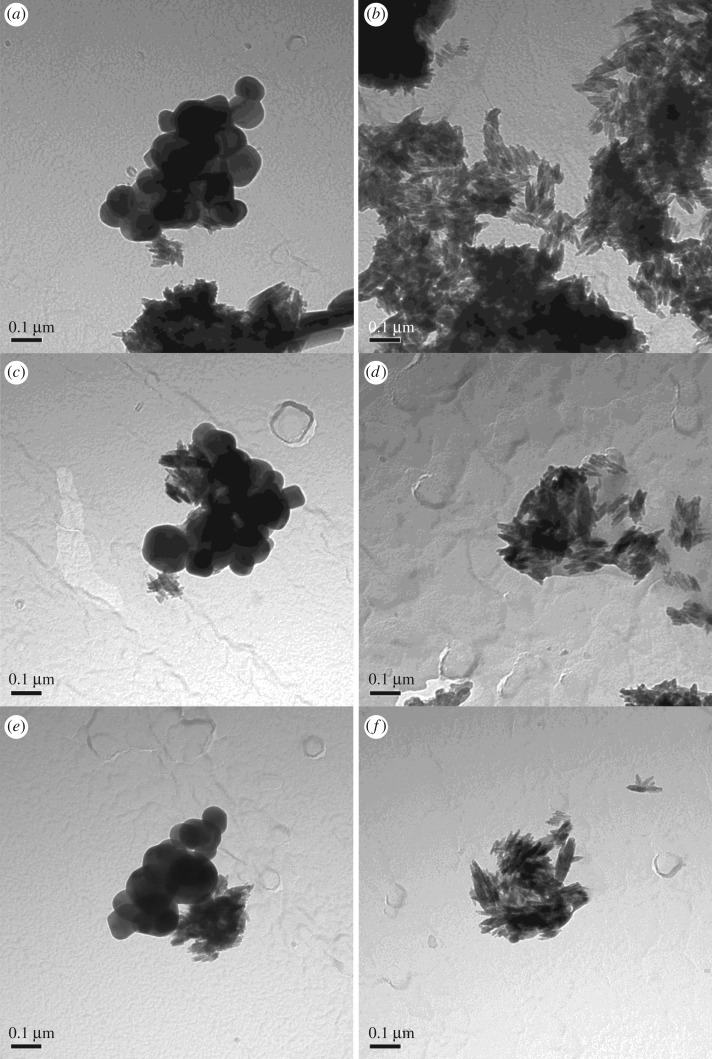
TEM images at 150 000× at 80 kV of the complete formula. (*a*) Sample BAM-13A extracted with ethanol (68 ± 25 nm) and (*b*) sample BAM-13B extracted with ethanol (73 ± 23 nm). (*c*) BAM-13A extracted with methanol (58 ± 17 nm) and (*d*) sample BAM-13B extracted with methanol (66 ± 27 nm). (*e*) Sample BAM-13A extracted with acetone (72 ± 27 nm) and (*f*) sample BAM-13B extracted with acetone (59 ± 15 nm).

Table 1 displays the characteristics of the simple and complete sunscreen formulae obtained from the NanoDefine consortium (BAM-13A and BAM-13B). Three solvent extraction methods were performed in order to remove the TiO_2_ NMs from the surrounding compounds found in the formulated samples [3]. The extracted titania particles were then characterized. The individual particle size distributions (TEM) from the various extraction conditions agreed well for each process for both samples BAM-13A and BAM-13B, producing size ranges within the standard deviation of the measurements. TEM imaging (figure 1*a*,*c*,*e*) identified sample BAM-13A to have mixed morphology containing both rods and semi-spherical-shaped NMs sized between 66 ± 23 nm on average. The two morphologies (rods and semi-spheres) may be attributable to at least two types of TiO_2_ NMs (and possibly also the iron oxide used for pigmentation) present in the complete formula.

**Table 1.** Characterization of commercial, complete and simple formula TiO_2_ samples. n.d., not determined.

sample	crystalline structure (XRD)	morphology (TEM)	size by TEM (nm)^a^	composition	BET surface area (m^2^ g^−1^)
TTO NJE8 (commercial)	rutile	rods	92 ± 22	TiO_2_, jojoba esters, alumina	772.57
JTTO MS7 (commercial)	rutile	rods	79 ± 26	TiO_2_, methicone, alumina	1396.26
UV Balance Powder 100 NJE8 (commercial)	rutile	semi-spherical	W 44 (±11) × l 71 (±23)	TiO_2_, jojoba esters, alumina	25.82
TiO_2_ TA-100 (commercial)	rutile	semi-spherical	40 ± 13	TiO_2_, silica, alumina	37.42
BAM-13A: ethanol extracted (complete formula)	rutile and anatase	rods and semi-spherical	68 ± 25	TiO_2_, iron oxide, alumina, stearic acid	n.d.
BAM-13A: methanol extracted (complete formula)	rutile and anatase	rods and semi-spherical	58 ± 17		n.d.
BAM-13A: acetone extracted (complete formula)	rutile and anatase	rods and semi-spherical	72 ± 27		n.d.
BAM-13B: ethanol extracted (simple formula)	rutile	rods	73 ± 23	TiO_2_, alumina, stearic acid	n.d.
BAM 13B: methanol extracted (simple formula)	rutile	rods	66 ± 27		n.d.
BAM: 13B: acetone extracted (simple formula)	rutile	rods	59 ± 15		n.d.

^a^Size by TEM is based on the average of 100 particles.

**Figure 2.**
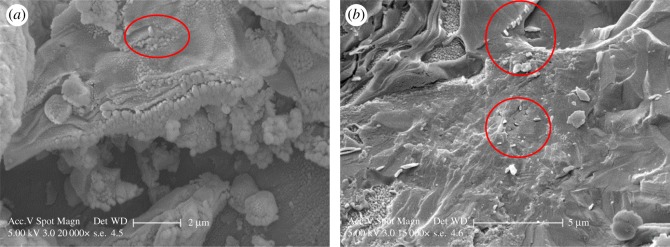
(*a*) Cryo-SEM images at 20 000× at 5 kV of the complete formula (untreated) sample BAM-13A and (*b*) cryo-SEM image at 15 000× at 5 kV of the untreated sample BAM-13B. NMs are indicated by the red circles.

Sample BAM-13B only contained rod-shaped NMs (figure 1*b*,*d*,*f*) that were also individually sized between 66 ± 21 nm. The TEM imaging shows some clustering of the NMs, with definitive outlines of individual particles to confirm loose agglomerates. Despite some ice contamination from the sample preparation, cryo-SEM imaging (figure 2*a*,*b*) revealed nanostructured features inside the complete and simple formula sunscreen residues, similar to the nanostructures observed from the TEM imaging.

**Figure 3.**
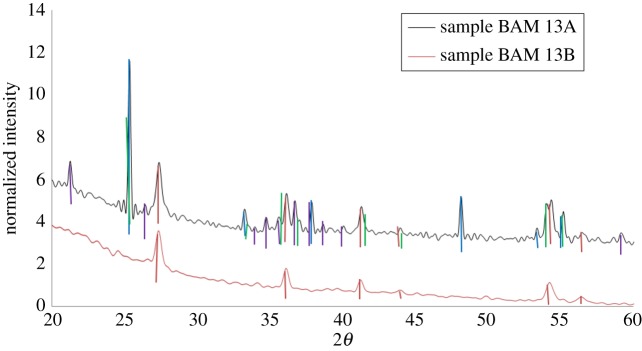
XRD spectra of complete formula (untreated). Sample A: BAM-13A. Diffraction peaks correspond to the standard data to show that the particles are composed of rutile (marked in red) (PDF 01-089-0553) and anatase (marked in blue) (PDF 04-006-9241) phase TiO_2_, with goethite iron oxide (marked in purple) (FeO(OH)) (PDF 04-015-2899) and with titanium aluminium oxide (marked in green) (PDF 04-011-8572). Sample B: BAM-13B. Diffraction peaks correspond to the standard data to show that the particles are composed of rutile (PDF 01-089-0553) phase TiO_2_ and titanium aluminium oxide (marked in green) (PDF 04-011-8572).

Structural analysis using XRD (figure 3) of the dried sunscreen lotion samples (no sample pretreatment) revealed that sample BAM-13A contained TiO_2_ of rutile crystalline structure with distinct diffraction peaks at 27°, 36°, 42° and 55° at 2*θ* [42] (marked by red in figure 3). TiO_2_ of anatase structure (marked in blue on figure 3) was identified with peaks at 25°, 37°, 38°, 39°, 48°, 54° and 55°. Iron oxide (goethite) was identified at peaks 21°, 26°, 33°, 34°, 35°, 37°, 38°, 40°, 41°, 43° and 59° (marked in purple). TiAl oxide was identified at peaks 26°, 36°, 39°, 41°, 44°, 54° and 57° (marked in green on figure 3). This information agrees with the mixed morphologies of NMs observed from the TEM and cryo-SEM imaging. On the other hand, sample BAM-13B contained only the rutile crystalline structure of TiO_2_ with the TiAl oxide coating, further complementing the morphological observations from the TEM imaging.

### 3.2. Characterization of commercially available powder TiO_2_ NMs

**Figure 4.**
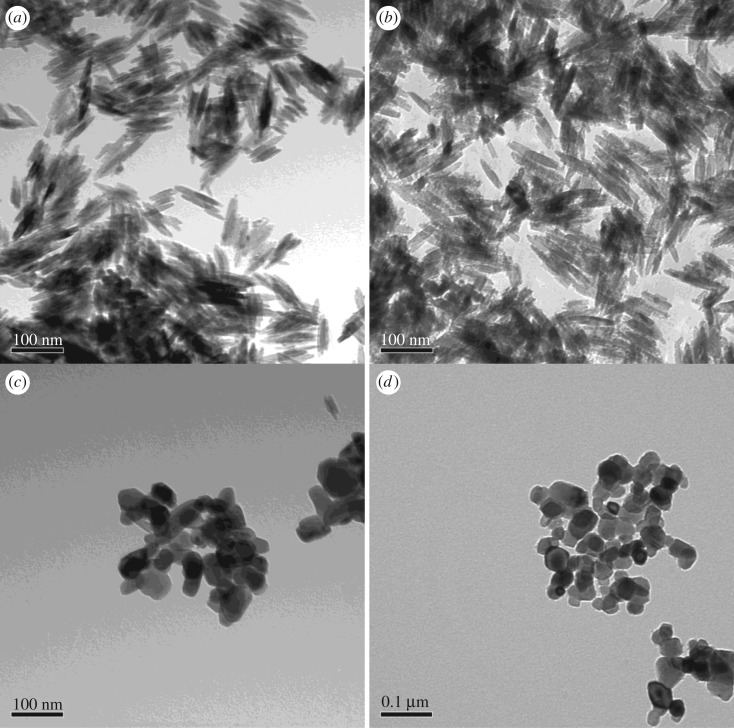
TEM images of the commercial TiO_2_ powders at 200 000× at 80 kV. (*a*) TTO NJE8, approximate size: 92 ± 22 nm, (*b*) JTTO MS7, approximate size: 79 ± 26 nm, (*c*) UV Balance Powder, approximate size: W 44 (±11) × l 71 (±23) nm and (*d*) TiO_2_ TA-100, approximate size: 40 ± 13 nm.

**Figure 5.**
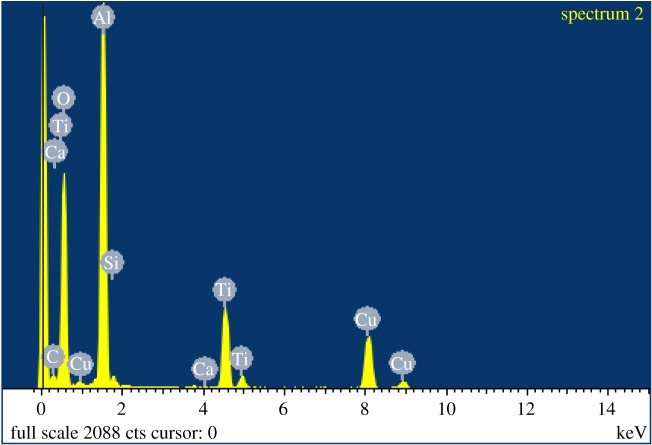
EDS spectrum of JTTO MS7 commercial powder TiO_2_ sample, showing the presence of TiO_2_, silica: possibly linked to the methicone surface surfactant and calcium from the powder sample. Copper and carbon are both from the copper–carbon film TEM grid used to mount the samples.

The characterization data for the commercially available TiO_2_ are given in table 1. TEM imaging revealed that commercial powdered TiO_2_ samples were morphologically similar for samples TTO NJE8 and JTTO MS7 (figure 4*a*,*b*), showing individual rod structures of smaller (79 ± 26 nm) and larger sizes (92 ± 22 nm) loosely agglomerated (possibly due to sample preparation). These rod structures were comparable to those observed from samples BAM-13A and BAM-13B for the TiO_2_ NMs. The UV Balance Powder 100 NJE8 and the TiO_2_ TA-100 samples, on the other hand, had a similar semi-spherical morphology to sample BAM-13A (complete formula) with sizes of 44 (±11) × 71 (±23) nm and 40 ± 13 nm (figure 4*c*,*d*). The corresponding EDS spectra (figure 5) confirmed that the samples contain Ti and Al. Note that the copper (Cu) and carbon (C) are background readings from the Cu–C film TEM grid used to mount the sample. The surface area analysis (table 1) also confirmed that TTO NJE8 and JTTO MS7 were similar with surface areas of 777.57 and 1396.26 m^2^ g^−1^ (table 1). In comparison, the UV Balance Powder 100 NJE8 and the TiO_2_ TA-100 samples had smaller surface areas at 25.82 and 37.42 m^2^ g^−1^ (table 1).

**Figure 6.**
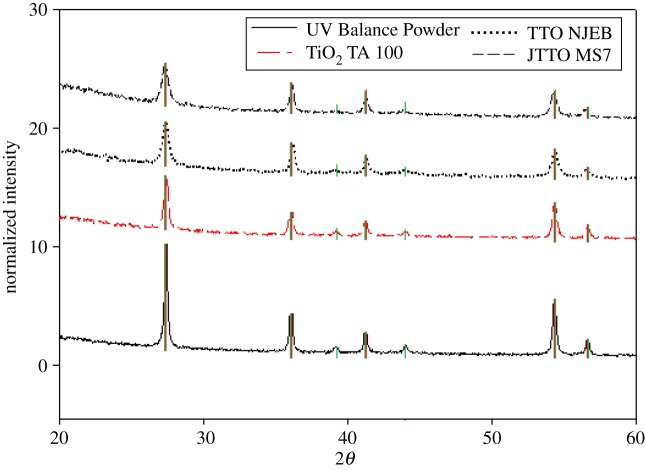
XRD diffraction patterns of the commercial TiO_2_ powders (from top to bottom trace). All diffraction peaks correspond to the rutile crystalline (marked in red) structure of titanium oxide and, additionally, titanium alumina oxides (marked in green), with correspondence to the standard pattern PDF 01-089-0552.

The XRD patterns of the commercial powder preparations containing the TiO_2_ NMs are shown in figure 6. The diffraction patterns at a normalized intensity all show strong peaks at 27°, 36° and 55°, agreeing with literature-cited sources for TiO_2_ nanostructures in the rutile phase [42]. Although subtle, additional peaks identified at 39°, 44° and 57° are conformational of the Al oxide layer coating the particle surfaces (as highlighted in green, figure 6) [1] and agree with the EDS findings (figure 5). Note that the iron oxide was not observed in any of the four commercial TiO_2_ powder samples analysed, when compared with the samples observed in §3.2 for the complete formulated sunscreen sample BAM-13A. This is due to the iron oxide (non-nano) being incorporated as a colouring agent in the complete formula at a later production stage.

**Figure 7.**
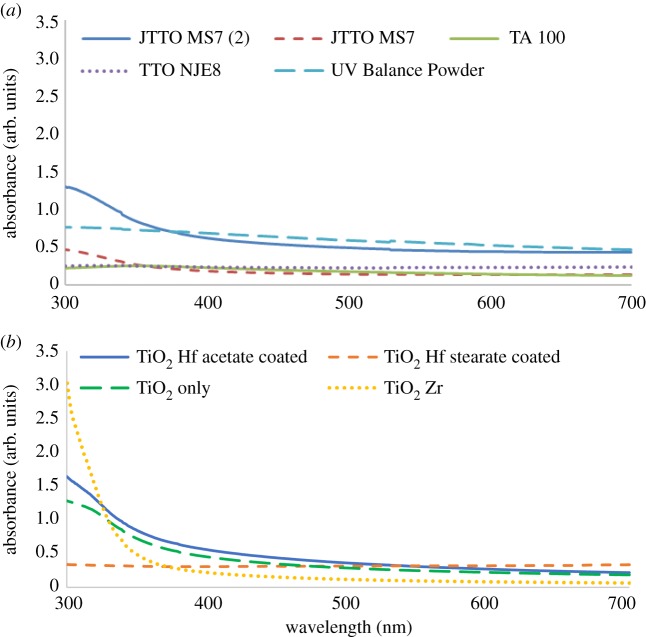
(*a*) UV–vis spectra for the commercially available TiO_2_ NMs and (*b*) UV–vis spectra of the synthesized doped TiO_2_ NMs.

The UV–vis spectra of the commercially available NMs demonstrated the typical pattern produced for TiO_2_ NMs for the JTTO MS 7 NM (figure 7*a*), the EBG of which is 2.89 eV. The rest of the available NMs (TA 100, TTO NJE8, UV Balance powder) presented a flat line, which is probably due to the low concentration of the available NMs that did not allow a proper signal to be acquired. It is interesting to note a small peak observed in all cases at approximately 530 nm, which can be attributed to the presence of impurities inside the structure of the materials. Similar peaks have been observed in the case of Ru [43] and Au [44] TiO_2_ composites.

The commercial manufactured powder TiO_2_ samples had additional surface treatments; this information was provided in the delivery notes of the samples and not obtained in house. For samples TTO NJE8 and UV Balance Powder 100 NJE8, the surface treatment was made up of jojoba esters, whereas JTTO MS7 was surface-treated with methicone, and TiO_2_ TA-100 was surface-treated with silica (exact ratios were not provided by the manufacturers). The alumina coating is designed to prevent catalytic properties, such as the formation of oxidative species or free radicals on the surface of the TiO_2_, while the hydrophobic organic coating improves the dispersion and stability of the NMs in oil/water emulsion of the formula [1,37].

Overall, all commercial TiO_2_ powder particles for use in sunscreen products investigated in this study contained NM properties as defined by the European Commission and the ISO, having size distributions that fall between 1 and 100 nm [45]. The powder TiO_2_ NMs provided sufficient structure, morphology and size data to support the design of the TiO_2_ and ZrO_2_ reference NMs.

### 3.3. Characterization of synthesized TiO_2_ and ZrO_2_ doped for reference materials

Based on the characterization data of commercially available NMs and those in simple and complete sunscreen formulae, we now report the synthesis of a catalogue of crystalline anatase and rutile TiO_2_ and ZrO_2_ NMs in a range of sizes and surface coatings to mimic those commercially available. The characterization data are summarized in table 2.

**Table 2.** Particles for reference materials/labelling and identification.

sample	crystalline structure (XRD)	morphology (TEM)	size nm (TEM)	composition	BET total specific surface area (m^2^ g^−1^)	concentration mg l^−1^ ICP-OES
TiO_2_	rutile	rods	30 ± 10 nm	TiO_2_	119.18	32
TiO_2_– alumina film-coated	rutile	rods	40 ± 11	TiO_2_, alumina	106.61	32
TiO_2_– Hf doped	anatase	semi-spherical	30 (±10) × 12 (±2)	TiO_2_, Hf, acetate	84.885	12
TiO_2_–Hf doped	anatase	semi-spherical	20 (±12) × 8 (±2)	TiO_2_, Hf, stearate	82.543	12
TiO_2_– Zr-doped	anatase	semi-spherical	42 (±10) × 12 (±3)	TiO_2_, Zr	80.452	10
ZrO_2_	monoclinic	mixed nanostructures	166 (±61) ± 46 (±17)	Zr	27.154	n.d.
ZrO_2_ doped with Hf	monoclinic	mixed nanostructures	166 (±49) × 54 (±17)	Zr, Hf	29.740	n.d.

#### 3.3.1. Labelled TiO_2_ reference materials

**Figure 8.**
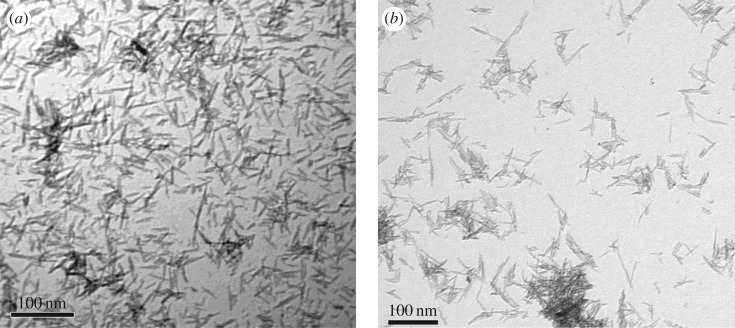
(*a*) TEM images of TiO_2_ particles for reference materials at 250 000×, approximate size 30 ± 10 nm, before the alumina coating process, and (*b*) TEM images at 200 000×, approximate size 40 ± 11 nm, after the alumina film coating process.

The hydrolysis and oxidation method of TiO_2_ synthesis produced rod-shaped NMs, as shown by TEM analysis, regardless of the presence or absence of the alumina coating (table 2 and figure 8). The TiO_2_ NMs coated with alumina show larger (40 ± 11 nm) and less aggregated particles than the TiO_2_ without an alumina coating (figure 8). The size increase observed confirms the successful attachment of the surface coating. The alumina coating process exploits the dissociation of the AlCl_3_, which is introduced in deionized water containing the TiO_2_ to form a hexaaquaaluminium complex [Al(H_2_O)_6_]^3+^ which transforms when the pH is adjusted with NaOH to produce Al_2_O_3_. The aluminium species is then adsorbed onto the TiO_2_ surface to form a coating around the particle surface, resulting in increased size, as observed [37].

**Figure 9.**
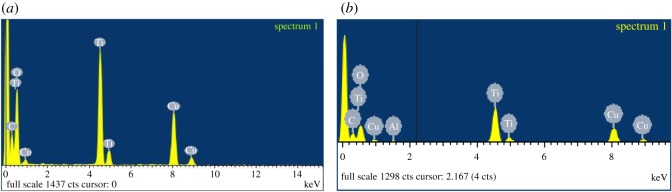
EDS spectrum of TiO_2_ particles for reference materials. (*a*) Pure TiO_2_ particles before alumina film coating and (*b*) TiO_2_ particles after alumina film coating, showing the presence and absence of the aluminium peak.

The morphological appearance of the alumina-coated TiO_2_ particles matched well the NJE8 and JTTO MS7 commercial samples (figure 4*a*,*b*), and the NMs observed in both samples BAM-13A and BAM-13B of the complete and simple sunscreen formulae (figure 1). EDS analysis in figure 9*a*,*b* provides information on the TiO_2_ particles before and after the coating process. The presence of Al after the coating process is comparable to the EDS presented in figure 5 for the commercially available powder TiO_2_ NMs, further confirming the presence of the alumina surface treatment on the in-house synthesized particles.

**Figure 10.**
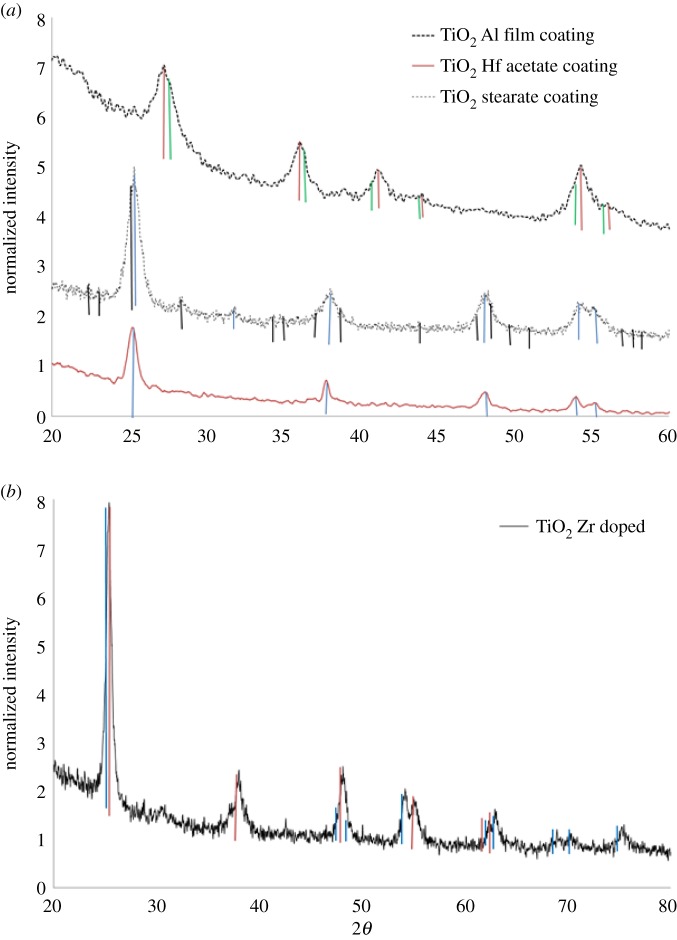
XRD diffraction patterns for TiO_2_ for reference materials. (*a*) Rutile (marked in red) TiO_2_ surface treated with titanium alumina oxide layer (marked in green) (PDF 00-014-0451), compared with anatase TiO_2_ (marked in blue) (PDF 01-086-1157) Hf-doped (marked in black) (PDF 01-089-0555) surface functionalized with stearate and acetate (top, middle and bottom trace, respectively). (*b*) XRD pattern for the anatase TiO_2_ (red) (PDF 01-086-1157) Zr-doped (blue) (PDF 04-013-9933 Zr oxide) particles.

In likeness to the commercially available powder NMs, the XRD results (figure 10*a*) confirm that the TiO_2_ particles with the alumina film coating have diffraction peaks at 27°, 36° and 55°, which agree with literature sources for TiO_2_ nanostructure in the rutile phase [42]. Additional peaks at 39°, 44° and 55°–57° confirmed the presence of the alumina coating as observed in the XRD spectra for the commercially available powder TiO_2_ particles (figure 4) (and confirms the EDS results; figure 9*b*). The Rietveld refinement of the TiO_2_ aluminium oxide-coated NMs is presented in table 3. The unit cell dimensions and properties are in good agreement with those of the reference phase, and the reduction in volume and density can be explained by the lower crystallinity of the synthesized material. Additionally, no Al incorporation into the crystal structure of the TiO_2_ was detected, which means that the Al is only present as a surface modification to the TiO_2_ NM core.

**Table 3.** Rieveld refinements.

		atomic coordinates				unit cell dimensions (Å)		angle (^o^)	unit cell properties	
material	atom	*x*	*y*	*z*	fraction	*α *= *b*	*c*	*β*	volume (Å^3^)	density (g cm^−3^)
TiO_2_ reference for rutile	Ti^4+^	0	0	0	n.a.	4.593	2.961	90	62.464	4.248
	O^2−^	0.305	0.305	0	n.a.					
TiO_2_–alumina film-coated	Ti^4+^	0	0	0	n.a.	4.599	2.949	90	62.390	4.253
	O^2−^	0.292	0.292	0	n.a.					
TiO_2_ reference for anatase	Ti^4+^	0.00	0.250	0.375	n.a.	3.771	9.430	90	134.099	3.957
	O^2−^	0.00	0.250	0.166	n.a.					
TiO_2_–Hf-doped acetate surface-coated (Ti_*x*_Hf_*y*_O_2 _: anatase)	Ti^4+^	0.00	0.250	0.375	0.95	3.753	8.758	90	123.332	4.582
	Hf^4+^	0.00	0.250	0.212	0.05					
	O^2−^	0.00	0.250	0.375	n.a.					
TiO_2_–Hf-doped, stearate surface-coated (Ti_*x*_Hf_*y*_O_2 _: anatase)	Ti^4+^	0.00	0.250	0.375	0.97	3.803	9.502	90	137.433	4.509
	Hf^4+^	0.00	0.250	0.153	0.03					
	O^2−^	0.00	0.250	0.375	n.a.					
TiO_2_–Zr-doped (Ti_*x*_Zr_*y*_O_2 _: anatase)	Ti^4+^	0.00	0.25	0.375	0.91	3.769	9.437	90	134.061	4.143
	Zr^4+^	0.00	0.25	0.163	0.09					
	O^2−^	0.00	0.25	0.375	n.a.					
ZrO_2_ reference	Zr^4+^	0.276	0.040	0.209	n.a.	5.141	5.322	99.1	140.635	5.820
	O^2−^	0.449	0.758	0.476	n.a.					
ZrO_2_–Hf-doped (Zr_*x*_Hf_*y*_O_2_)	Zr^4+^	0.276	0.040	0.286	0.98	5.163	5.335	99.6	141.180	5.573
	Hf^4+^	0.274	0.041	0.205	0.02					
	O^2−^	0.456	0.749	0.466	n.a.					

When analysed by BET in the absence of the Al oxide layer, the surface area was 119.18 m^2^ g^−1^ (table 2). In the presence of the alumina layer, the surface area was reduced slightly to 106.61 m^2^ g^−1^, which is consistent with increased particle size in the presence of the surface coating modification (table 2).

**Figure 11.**
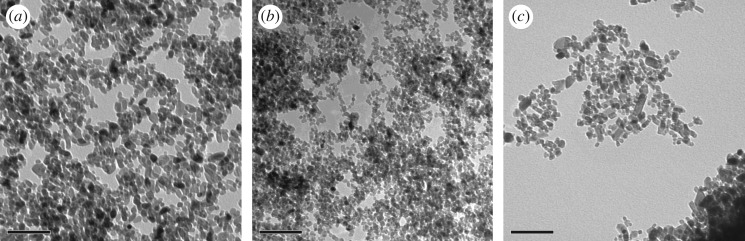
TEM images of the TiO_2_ reference materials at 250 000× at 80 kV. (*a*) Acetate surface functionalized TiO_2_ Hf doped, measured particle size: 30 (±10) × 12 (±2) nm, (*b*) sterate surface functionalized TiO_2_ Hf doped, measured particle size: 20 (±12) × 8 (±2) nm and (*c*) TiO_2_ Zr doped (no surface functionalization), measured particle size: 42 (±10) × 12 (±3) nm. Scale bars represent 100 nm.

On the contrary, synthesized TiO_2_ NMs with doping agents Hf and Zr produced by hydrothermal methods formed semi-spherical-shaped NMs (table 2 and figure 11*a*–*c*), which were similar to those observed for the UV Balance Powder and TiO_2_ TA-100 commercial powder samples (figure 4*c*,*d*) and those reported in the literature [41]. The addition of acetate and stearate was also used as a surface treatment to produce a hydrophobic layer, similar to the commercially available NMs and complete formula NMs (BAM-13a) (table 1). The adsorption of stearate and acetate forms a strong bidentate bond between the TiO_2_ surface and the carboxylic acid COOH group [41,46].

When TiO_2_ is doped with other metals, its properties are modified, including the crystalline phase. Diffraction peaks at 25°, 37° and 48° (figure 10*a*,*b*) in the literature indicate that the synthesized TiO_2_ NMs with doping agents Hf and Zr have an anatase crystalline phase [47] in comparison with the rutile rods produced previously by the hydrolysis and oxidation method. For the Hf-doped TiO_2_, the diffraction peak at 37° had drifted slightly to 38°, which is consistent with changes in the Ti crystalline lattice [48,49]. The ionic radii of Hf (IV) and Zr (IV) are 72 and 80 pm, respectively, and are both larger than that of Ti (IV), which is 56 pm. Therefore, substitutions of Ti positions in the crystal lattice by Hf and Zr ions will increase the size of the resulting NMs and will also cause slight drifts of the XRD diffraction peaks towards lower angles [48]. The XRD patterns also confirmed the addition of the doping agents using the cited literature and the diffraction patterns in the XRD Eva software database. The Hf-doped TiO_2_ NMs had additional diffraction peaks at 22°, 23°, 29°, 32°, 34°, 44°, 47°–49°, 51° and 57°–59° (marked in black in figure 10*a*), which were absent when compared with the commercial TiO_2_ samples and the complete formula TiO_2_ samples (figures 3 and 6). Furthermore, longer scans between 20° and 80° 2*θ* were required to obtain all corresponding peaks for the TiO_2_–Zr-doped particles at 30°, 53°, 55°, 62°, 70° and 75° (figure 10*b*) [48]. The additional diffraction peaks for the TiO_2_–Zr NMs were not observed in the Hf-doped TiO_2_ or the commercial and simple/complete sunscreen formula TiO_2_ preparations.

Rietveld refinement of the TiO_2_ anatase NM, doped with either Hf or Zr, showed a good agreement and low error between the measured XRD diffractograms and the model produced through GSAS II, despite the low crystallinity of the NMs. The results suggest that both Hf and Zr were incorporated in the TiO_2_ structure with respective changes in the unit cell properties and atomic coordinates (table 3), which can be explained by the difference in the ionic radii of those elements (Ti: 147 pm; Hf: 159 pm; and Zr: 160 pm). In the case of the Hf-doped TiO_2_, approximately 5% and 3% of Hf was incorporated inside the TiO_2_ structure and their chemical formulae correspond to Ti_0.95_Hf_0.5_O_2_ and Ti_0.97_Hf_0.03_O_2_ for the acetate- and the stearate-coated NMs, respectively. The higher- and lower-doped TiO_2_ led to a less and more crystalline material, respectively, when compared with the unlabelled NMs, as the changes in the *α* and *c* dimensions and unit cell volume (table 3) suggest, which can also be observed through the diffractograms, which present either broader or sharper peaks for the acetate- and stearic acid-coated NMs, respectively. Similarly, doping with 9% of Zr (Ti_0.91_Zr_0.09_O_2_) and no surface treatment led to an NM, which is slightly less crystalline than the unlabelled NMs, which suggests that the crystallinity of the NM is affected by both the dopant and the potential surface treatment, which prevents NMs from growing [37].

When analysed by BET, the Hf-labelled TiO_2_ NMs had a total surface area (table 2) of 84.885 m^2^ g^−1^ (acetate surface-treated) and 82.543 m^2^ g^−1^ (stearate surface-treated). The TiO_2_–Zr-doped NMs had a similar surface of 80.542 m^2^ g^−1^. When compared with the commercially available NMs (table 1), they were considerable smaller than the rod-shaped NMs and had around double the surface area of the UV Balance Powder 100 NJE8 and TiO_2_ TA-100 samples of similar morphology.

**Figure 12.**
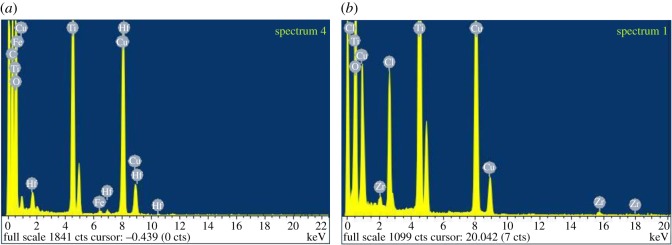
EDS spectrum of TiO_2_ particles for reference materials. (*a*) Corresponding to TiO_2_ particles with the Hf dopant, as shown by the presence of the Hf peaks. (*b*) Corresponding to particles with the Zr dopant, as shown by the presence of the Zr peaks.

It can be seen in figure 11*a*,*b* that the NMs' diameters are 30 (±10) × 12 (±2) nm for the TiO_2_–Hf doped with acetate surface treatment and 20 (±12) × 8 (±2) nm for the stearate surface-treated TiO_2_–Hf particles. Figure 10*c* shows that the TiO_2_–Zr-doped NMs without surface treatment are larger at 42 (±10) × 12 (±3) nm. Therefore, the type of doping agent influences NM size, as has surface treatment. There were no morphological shape differences between the different surface-treated TiO_2_–Hf-doped NMs and the TiO_2_–Zr-doped NMs without surface treatment. For confirmation of the Hf and Zr doping, figure 12*a* reports the EDS spectra for TiO_2_ NMs with the presence of Hf and figure 11*b* confirms the presence of the Zr dopant. XRD patterns (figure 10*a*,*b*) produced line broadening diffraction peaks, which are due to the nanosized nature of the samples and are consistent with other literature findings [41].

The UV–vis spectra of the doped TiO_2_ NMs can be seen in figure 7*b*. The pure TiO_2_ NM demonstrated an absorption peak at approximately 313 nm and had an EBG of 3.14 eV. The difference between this value and that calculated for the commercial JTTO MS 7 NM (2.89 eV) is due to the differences in the anatase and rutile crystal structures. These values are also similar to those reported in the literature of 3.3 and 3.0 eV for anatase and rutile structures [50], respectively, and the differences can be attributed to the different synthesis protocols and potential impurities present during production. Doping the TiO_2_ NMs with Zr (TiO_2_ Zr) resulted in a shift of the observed spectrum towards the lower wavelengths and the UV part of the spectrum, which signifies decreased photocatalytic activity. This is also supported by the calculated EBG of 3.61 eV. On the other hand, doping with Hf (TiO_2_ Hf acetate-coated) resulted in a shift of the observed spectrum towards the visible part of the spectrum and EBG of 3.13 eV. To compare with the commercial NMs, the TiO_2_ Hf (stearate-coated) was too dilute to measure. The shift towards the visible part of the wavelength spectrum and the reduction in EBG for the Hf-doped NMs also signify increased photocatalytic activity for the synthesized NMs, which can be explained by the presence of a new energy level in the band gap of the TiO_2_ NM, due to the presence of other metal elements [20,51]. These new energy levels are more able to absorb photons and increase the number of excited electrons in the conduction and holes in the valence band [20,51]. The production of potentially damaging radicals can also take place, especially for pure anatase TiO_2_ NMs due to their smaller size, when compared with rutile TiO_2_, and the overlapping of its conduction band with the redox potential of biological reactions that can lead to oxidative stress [52,53].

On the other hand, HfO_2_ NMs are less likely to produce ROS, and thus oxidative stress, due to the lack of overlap with the redox potential [52], which is further supported by the lack of toxic observations during *in vitro* experiments [27,28]. As a result, the use of Hf as a doping agent can decrease the toxic potential of TiO_2_ NMs, while enhancing its photocatalytic activities, thus making safer the use of such NMs in cosmetics. Doping with Zr, on the other hand, resulted in a shift towards the UV part of the spectrum and increases in the EBG, which signifies a decrease in the photocatalytic activity of TiO_2_, although such NMs could be useful in applications requiring the filtering of UV light, i.e. windows filters. At the same time, the conduction band of ZrO_2_ NMs is closer to the redox potential range, which means that any reduction in the toxicity potential of TiO_2_ NMs will be lower than that for Hf. This is further supported by the moderate toxicity observed during *in vitro* experiments [26]. In any case, further study is needed, including toxicity experiments, to verify this safer-by-design approach that can also act as a tracer for the risk assessment of TiO_2_ NMs.

#### 3.3.2. Labelled ZrO_2_ reference materials

**Figure 13.**
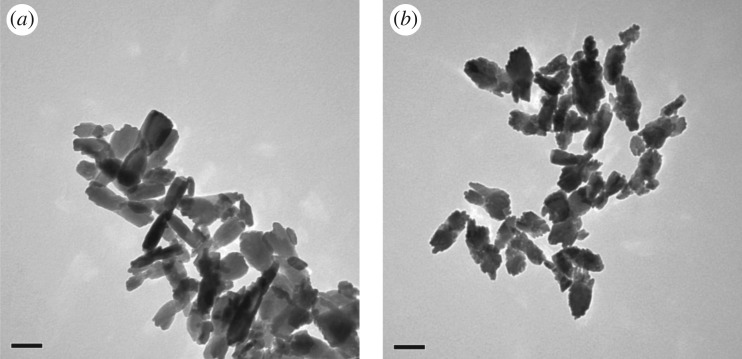
(*a*) TEM images at 150 000× at 80 kV of the ZrO_2_ NMs and (*b*) TEM image at 150 000× at 80 kV of ZrO_2_ doped with Hf for reference materials. Scale bars represent 100 nm.

**Figure 14.**
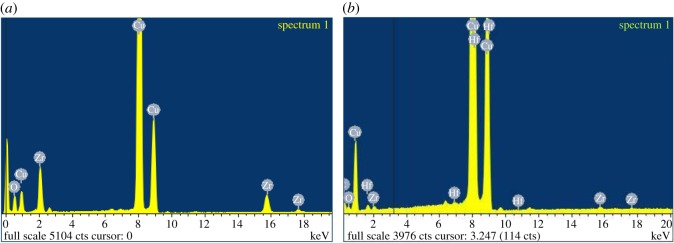
EDS spectrum of ZrO_2_ particles for reference materials. (*a*) Pure ZrO_2_ particles with no other peaks present and (*b*) Hf-doped ZrO_2_ as shown by the presence of Hf.

Labelled ZrO_2_ NMs were also synthesized in addition to the TiO_2_ NMs (figure 13*b*). The average size of the ZrO_2_ NMs containing Hf are 132 (±30) × 57 (±14) nm based on 100 particle counts (table 2). Morphological observations (figure 13) show that the majority of the ZrO_2_ NMs are much larger than those prepared with the Ti precursor and are morphologically different from each other. Supplementary to the TEM data, EDS measurements (figure 14*a*) show the ZrO_2_ NMs synthesized are absent of the Hf dopant, and with the addition of the Hf dopant (figure 14*b*). The measurements show that the presence of Zr accounts for approximately 98.87% for Zr, 0.16% for oxygen and 0.97% for Hf (atomic percentages).

**Figure 15.**
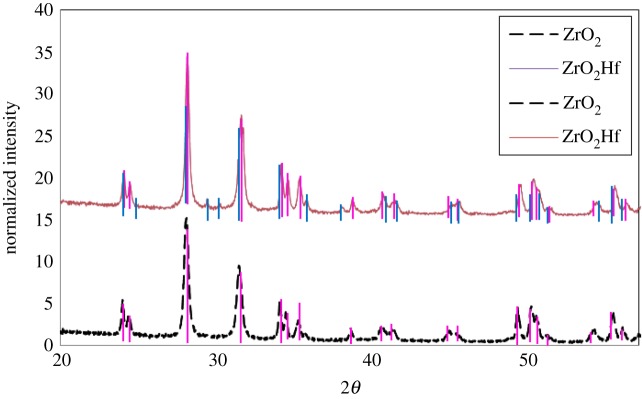
XRD spectrum of ZrO_2_- (pink) (PDF 00-037-1484) and Hf-doped ZrO_2_ (blue) (PDF 04-017-5852) particles (top and bottom trace, respectively). Both producing monoclinic crystalline structures.

Furthermore, the XRD spectra in figure 15 show that the ZrO_2_ NMs without a Hf dopant have a monoclinic crystalline structure, corresponding to a standard pattern (PDF: 00-037-1484) and previous literature [29,54]. Although the XRD spectra for the doped ZrO_2_ NMs are very similar to those without Hf, subtle differences show that the ZrO_2_ NMs containing the Hf dopant have additional diffraction peaks at 29°, 30° and 38° at 2*θ*. According to the standard data PDF 01-075-3557, the particles are composed of Zr–Hf oxide with a monoclinic crystalline structure, agreeing with the EDS results (figure 14).

The presence of Hf inside the ZrO_2_ structure was again verified through Rietveld refinement. In this case (table 3), the amount of Hf incorporated inside the Zr oxide structure was 2% (Zr_0.98_Hf_0.02_O_2_) and led to an increase in the unit cell dimensions and, subsequently, crystallinity, when compared with the undoped material. This increase is opposite to that observed for the Zr-doped TiO_2_, which also had no surface treatment, and further supports the suggestion that both the ionic radius of the dopant and the surface treatment affect the degree of crystallinity of an NM. The difference in this case possibly lies in the similar ionic radii of Zr and Hf (160 and 159, respectively).

## 4. Conclusion

The aim of this study was to test a safer-by-design approach for the production of labelled TiO_2_ NMs, which will also lead to increased NM traceability and assist exposure, fate and risk assessments. In recent years, research into the fate and behaviour of such TiO_2_ NMs from sunscreen and industrial products has relied on either solvent extraction from commercially available products or nanopowders [30], such as those referenced in this paper. However, these products are often difficult to trace in environmental matrices, and thus, accurate results are difficult to obtain, and concentrations are hard to distinguish from naturally occurring titania and NM titania. We compared the size, morphology, surface area and crystalline structures of several TiO_2_-containing products from complete formulae to simple nanopowders for use in commercial products. We used these inputs to influence the successful synthesis of a library of particles, which can be used as reference materials comparable to the TiO_2_ used in sunscreen and industrial formulae and can be distinguished from background concentrations of titania in environmental matrices, cosmetics and food.

In summary, this paper reports a simple hydrolysis and oxidation method of TiO_2_ synthesis, which successfully produced rod-shaped rutile phase particles, both with and without Al oxide coating and similar to nano-TiO_2_ found in commercial sunscreen formulae. The novel hydrothermal method also successfully produced TiO_2_ and ZrO_2_ NMs, as well as Hf- and Zr- doped variants, each of which were shown to be structurally unique phases. Given that Hf and Zr are both relatively rare in the environment, the NMs presented in this paper can be uniquely identified in complex matrices and therefore serve as tracers in either laboratory or environmental studies.

## Data accessibility

There are no additional data to accompany this manuscript. All relevant datasets are within the main body of the manuscript. All protocols and software used are stated fully in the methodology section. There are no coding lists for this research.
